# Supplementary material for: Dietary Fibre Intake in Relation to Asthma, Rhinitis and Lung Function Impairment—A Systematic Review of Observational Studies
Source: Nutrients. 2021 Oct 14;13(10):3594. doi: 10.3390/nu13103594 (PMC8539618; doi:10.3390/nu13103594)
Supplement: Supplementary file 1 [file nutrients-13-03594-s001.zip › nutrients-1411867-SI.pdf]

Table S1. Completed PRISMA checklist

PRISMA 2020 Checklist

Section and Topic	Item #	Checklist item	Location where item is reported
**TITLE**			
Title	1	Identify the report as a systematic review.	Title
**ABSTRACT**			
Abstract	2	See the PRISMA 2020 for Abstracts checklist.	Abstract
**INTRODUCTION**			
Rationale	3	Describe the rationale for the review in the context of existing knowledge.	Introduction
Objectives	4	Provide an explicit statement of the objective(s) or question(s) the review addresses.	Introduction
**METHODS**			
Eligibility criteria	5	Specify the inclusion and exclusion criteria for the review and how studies were grouped for the syntheses.	2.2
Information sources	6	Specify all databases, registers, websites, organisations, reference lists and other sources searched or consulted to identify studies. Specify the date when each source was last searched or consulted.	2.3, 2.4
Search strategy	7	Present the full search strategies for all databases, registers and websites, including any filters and limits used.	2.4,supplement
Selection process	8	Specify the methods used to decide whether a study met the inclusion criteria of the review, including how many reviewers screened each record and each report retrieved, whether they worked independently, and if applicable, details of automation tools used in the process.	2.5
Data collection process	9	Specify the methods used to collect data from reports, including how many reviewers collected data from each report, whether they worked independently, any processes for obtaining or confirming data from study investigators, and if applicable, details of automation tools used in the process.	2.6
Data items	10a	List and define all outcomes for which data were sought. Specify whether all results that were compatible with each outcome domain in each study were sought (e.g. for all measures, time points, analyses), and if not, the methods used to decide which results to collect.	2.6
	10b	List and define all other variables for which data were sought (e.g. participant and intervention characteristics, funding sources). Describe any assumptions made about any missing or unclear information.	2.6
Study risk of bias assessment	11	Specify the methods used to assess risk of bias in the included studies, including details of the tool(s) used, how many reviewers assessed each study and whether they worked independently, and if applicable, details of automation tools used in the process.	2.7
Effect measures	12	Specify for each outcome the effect measure(s) (e.g. risk ratio, mean difference) used in the synthesis or presentation of results.	
Synthesis methods	13a	Describe the processes used to decide which studies were eligible for each synthesis (e.g. tabulating the study intervention characteristics and comparing against the planned groups for each synthesis (item #5)).	2.8
	13b	Describe any methods required to prepare the data for presentation or synthesis, such as handling of missing summary statistics, or data conversions.	2.8
	13c	Describe any methods used to tabulate or visually display results of individual studies and syntheses.	2.8
	13d	Describe any methods used to synthesize results and provide a rationale for the choice(s). If meta-analysis was performed, describe the model(s), method(s) to identify the presence and extent of statistical heterogeneity, and software package(s) used.	2.8
	13e	Describe any methods used to explore possible causes of heterogeneity among study results (e.g. subgroup analysis, meta-regression).	
	13f	Describe any sensitivity analyses conducted to assess robustness of the synthesized results.	
Reporting bias assessment	14	Describe any methods used to assess risk of bias due to missing results in a synthesis (arising from reporting biases).	
Certainty assessment	15	Describe any methods used to assess certainty (or confidence) in the body of evidence for an outcome.	2.9

PRISMA 2020 Checklist

Section and Topic	Item #	Checklist item	Location where item is reported
**RESULTS**			
Study selection	16a	Describe the results of the search and selection process, from the number of records identified in the search to the number of studies included in the review, ideally using a flow diagram.	3.1, figure 1
	16b	Cite studies that might appear to meet the inclusion criteria, but which were excluded, and explain why they were excluded.	3.1, discussion
Study characteristics	17	Cite each included study and present its characteristics.	3.2, table 1
Risk of bias in studies	18	Present assessments of risk of bias for each included study.	3.4
Results of individual studies	19	For all outcomes, present, for each study: (a) summary statistics for each group (where appropriate) and (b) an effect estimate and its precision (e.g. confidence/credible interval), ideally using structured tables or plots.	3.3, table 1
Results of syntheses	20a	For each synthesis, briefly summarise the characteristics and risk of bias among contributing studies.	figure 2
	20b	Present results of all statistical syntheses conducted. If meta-analysis was done, present for each the summary estimate and its precision (e.g. confidence/credible interval) and measures of statistical heterogeneity. If comparing groups, describe the direction of the effect.	
	20c	Present results of all investigations of possible causes of heterogeneity among study results.	
	20d	Present results of all sensitivity analyses conducted to assess the robustness of the synthesized results.	
Reporting biases	21	Present assessments of risk of bias due to missing results (arising from reporting biases) for each synthesis assessed.	
Certainty of evidence	22	Present assessments of certainty (or confidence) in the body of evidence for each outcome assessed.	3.5
**DISCUSSION**			
Discussion	23a	Provide a general interpretation of the results in the context of other evidence.	Discussion
	23b	Discuss any limitations of the evidence included in the review.	Discussion
	23c	Discuss any limitations of the review processes used.	Discussion
	23d	Discuss implications of the results for practice, policy, and future research.	Discussion
**OTHER INFORMATION**			
Registration and protocol	24a	Provide registration information for the review, including register name and registration number, or state that the review was not registered.	2.1
	24b	Indicate where the review protocol can be accessed, or state that a protocol was not prepared.	
	24c	Describe and explain any amendments to information provided at registration or in the protocol.	
Support	25	Describe sources of financial or non-financial support for the review, and the role of the funders or sponsors in the review.	Back matter
Competing interests	26	Declare any competing interests of review authors.	Back matter
Availability of data, code and other materials	27	Report which of the following are publicly available and where they can be found: template data collection forms; data extracted from included studies; data used for all analyses; analytic code; any other materials used in the review.	

From: Page MJ, McKenzie JE, Bossuyt PM, Boutron I, Hoffmann TC, Mulrow CD, et al. The PRISMA 2020 statement: an updated guideline for reporting systematic reviews. BMJ 2021;372:n71. doi: 10.1136/bmj.n71

For more information, visit: http://www.prisma-statement.org/

Table S2. Medline search construct

**MeSH thesaurus terms**	**Free text terms**
Dietary fiber	Dietary fiber
	((fiber* or fibre* or roughage* or wheat bran*) and (cereal* or consum* or diet* or fruit* or intake or ingest* or nutrit* or vegetable*))
Lung Diseases, Obstructive	Lung Diseases, Obstructive
Rhinitis	Rhinitis
Respiratory Function Tests	Respiratory Function Tests
	(asthma* or bronchi* or COPD or cough* or emphym* or phlegm* or hay fever or rinit* or rhinoconjunctivitis or wheeze*)
	(obstructive (airflow or airway or lung or pulmonary))
	((expiratory or inspiratory or lung or maximal or pulmonary or respiratory or residual or vital) (airflow or capacit* or flow or ventilation or volume*))
	(spirometry or bronchospirometry)

Table S3. Documentation of search strategies

1. Medline

Interface: Ovid MEDLINE(R) and Epub Ahead of Print, In-Process & Other Non-Indexed Citations and Daily<br><br>Date of Search: 7th June 2021<br><br>Number of hits: 436<br><br>Comment: In Ovid, two or more words are automatically searched as phrases; i.e. no quotation marks are needed	Field labels<br><br>• exp/ = exploded MeSH term<br>• / = non exploded MeSH term<br>• .ti,ab,kf. = title, abstract and author keywords<br>• adjx = within x words, regardless of order<br>• * = truncation of word for alternate endings

1	Dietary Fiber/	17905
2	((fiber* or fibre* or roughage* or wheat bran*) and (cereal* or consum* or diet* or fruit* or intake or ingest* or nutrit* or vegetable*)).ti,ab,kf.	39656
3	or/1-2	45377
4	exp Lung Diseases, Obstructive/	218018
5	exp Rhinitis/	35428
6	exp Respiratory Function Tests/	239717
7	(asthma* or bronchi* or COPD or cough* or emphym* or hay fever or phlegm* or rinit* or rhinoconjunctivitis or wheeze*).ti,ab,kf.	359876
8	(obstructive adj3 (airflow or airway or lung or pulmonary)).ti,ab,kf.	62847
9	((expiratory or inspiratory or lung or maximal or pulmonary or respiratory or residual or vital) adj3 (airflow or capacit* or flow or ventilation or volume*)).ti,ab,kf.	108136
10	(spirometry or bronchospirometry).ti,ab,kf.	18664
11	or/4-10	677729
12	3 and 11	565
13	12 not (animals not humans).sh.	463
14	limit 13 to (english or french or german or swedish)	436

2. Embase

Interface: embase.com<br><br>Date of Search: 7 June 2021<br><br>Number of hits: 846<br><br>Comment: Emtree is the controlled vocabulary in Embase	Field labels<br><br>• /exp = exploded Emtree term<br>• /de = non exploded Emtree term<br>• ti,ab,kw = title, abstract and author keywords<br>• NEAR/x = within x words, regardless of order<br>• * = truncation of word for alternate endings

#1 'dietary fiber'/de
#2 (fiber*:ti,ab,kw OR fibre*:ti,ab,kw OR roughage*:ti,ab,kw OR 'wheat bran*':ti,ab,kw) AND (cereal*:ti,ab,kw OR consum*:ti,ab,kw OR diet*:ti,ab,kw OR fruit*:ti,ab,kw OR intake:ti,ab,kw OR ingest*:ti,ab,kw OR nutrit*:ti,ab,kw OR vegetable*:ti,ab,kw)
#3 #1 OR #2

#4 'obstructive lung disease'/exp
#5 'rhinitis'/exp
#6 'lung function test'/exp
#7 asthma*:ti,ab,kw OR bronchi*:ti,ab,kw OR copd:ti,ab,kw OR cough*:ti,ab,kw OR emphym*:ti,ab,kw OR 'hay fever':ti,ab,kw OR phlegm*:ti,ab,kw OR rinit*:ti,ab,kw OR rhinoconjunctivitis OR wheeze*:ti,ab,kw
#8 (obstructive NEAR/3 (airflow OR airway OR lung OR pulmonary)):ti,ab,kw
#9 ((expiratory OR inspiratory OR lung OR maximal OR pulmonary OR respiratory OR residual OR vital) NEAR/3 (airflow OR capacit* OR flow OR ventilation OR volume*)):ti,ab,kw
#10 spirometry:ti,ab,kw OR bronchospirometry:ti,ab,kw
#11 #4 OR #5 OR #6 OR #7 OR #8 OR #9 OR #10

#12 #3 AND #11

#13 #12 NOT ([animals]/lim NOT [humans]/lim) AND ([english]/lim OR [french]/lim OR [german]/lim OR [swedish]/lim)

3. Cochrane Library

Interface: Wiley<br><br>Date of Search: 7th June 2021<br><br>Number of hits: 80 (78 trials + 2 reviews)	Field labels<br><br>• ti,ab,kw = title, abstract and author keywords<br>• NEAR/x = within x words, regardless of order<br>• * = truncation of word for alternate endings

#1 ((fiber* or fibre* or roughage* or "wheat bran*") AND (cereal* or consum* or diet* or fruit* or intake or ingest* or nutrit* or vegetable*)):ti,ab,kw
#2 (asthma* or bronchi* or COPD or cough* or emphym* or "hay fever" or phlegm* or rinit* or rhinoconjunctivitis or wheeze*):ti,ab,kw
#3 (obstructive NEAR/3 (airflow or airway or lung or pulmonary)):ti,ab,kw
#4 ((expiratory or inspiratory or lung or maximal or pulmonary or respiratory or residual or vital) NEAR/3 (airflow or capacit* or flow or ventilation or volume*)):ti,ab,kw
#5 (spirometry or bronchospirometry):ti,ab,kw
#6 #2 or #3 or #4 or #5

#7 #1 AND #6

#8 #7 NOT (clinicaltrials or trialsearch):so

4. Web of Science Core Collection

Interface: Clarivate Analytics<br><br>Date of Search: 7th June 2021<br><br>Number of hits: 495	Field labels<br><br>• TS/Topic = title, abstract, author keywords and Keywords Plus<br>• NEAR/x = within x words, regardless of order<br>• * = truncation of word for alternate endings<br><br>Note: sometimes “quotation marks” are needed for single search terms to avoid automatic term mapping (lemmatization).

#1 TS=(((fiber* or fibre* or roughage* or "wheat bran*") and (cereal* or consum* or diet* or fruit* or intake or ingest* or nutrit* or vegetable*)))

#2 TS=((asthma* or bronchi* or COPD or cough* or emphym* or "hay fever" or phlegm* or rinit* or rhinoconjunctivitis or wheeze*)) OR TS=((obstructive NEAR/2 (airflow or airway or lung or pulmonary))) OR TS=(((expiratory or inspiratory or lung or maximal or pulmonary or respiratory or residual or vital) NEAR/2 (airflow or capacit* or flow or ventilation or volume*))) OR TS=((spirometry or bronchospirometry))

#3 #1 AND #2 Refined by: LANGUAGES: (ENGLISH OR FRENCH OR GERMAN)

5. Scopus

Interface: Elsevier<br><br>Date of Search: 7th June 2021<br><br>Number of hits: 458	Field labels<br><br>• TITLE-ABS = title and abstract<br>• AUTHKEY = author keywords<br>• W/ = within x words, regardless of order<br>• * = truncation of word for alternate endings

TITLE-ABS ((fiber* OR fibre* OR roughage* OR "wheat bran*") AND (cereal* OR consum* OR diet* OR fruit* OR intake OR ingest* OR nutrit* OR vegetable*)) OR AUTHKEY ((fiber* OR fibre* OR roughage* OR "wheat bran*") AND (cereal* OR consum* OR diet* OR fruit* OR intake OR ingest* OR nutrit* OR vegetable*))

AND

TITLE-ABS (asthma* OR bronchi* OR copd OR cough* OR emphym* OR "hay fever" OR phlegm* OR rinit* OR rhinoconjunctivitis OR wheeze*) OR AUTHKEY (asthma* OR bronchi* OR copd OR cough* OR emphym* OR "hay fever" OR phlegm* OR rinit* OR rhinoconjunctivitis OR wheeze*) OR TITLE-ABS (obstructive W/2 (airflow OR airway OR lung OR pulmonary)) OR AUTHKEY (obstructive W/2 (airflow OR airway OR lung OR pulmonary)) OR TITLE-ABS ((expiratory OR inspiratory OR lung OR maximal OR pulmonary OR respiratory OR residual OR vital) W/2 (airflow OR capacit* OR flow OR ventilation OR volume*)) OR AUTHKEY ((expiratory OR inspiratory OR lung OR maximal OR pulmonary OR respiratory OR residual OR vital) W/2 (airflow OR capacit* OR flow OR ventilation OR volume*)) OR TITLE-ABS (spirometry OR bronchospirometry) OR AUTHKEY (spirometry OR bronchospirometry)

AND

(LIMIT-TO (LANGUAGE , "English") OR LIMIT-TO (LANGUAGE , "German") OR LIMIT-TO (LANGUAGE , "French"))
